# A short-term price prediction-based trading strategy

**DOI:** 10.1371/journal.pone.0294970

**Published:** 2024-03-07

**Authors:** Weijia Fan, Ru Zhang, Hao He, Siyu Hou, Yongbo Tan

**Affiliations:** 1 School of Management, Harbin University of Commerce, Harbin, P. R. China; 2 Computer Vision Institute, College of Computer Science and Software Engineering, Shenzhen University, Shenzhen, P. R. China; 3 National Engineering Laboratory for Big Data System Computing Technology, Shenzhen University, Shenzhen, P. R. China; Nanjing University of Science and Technology, CHINA

## Abstract

Quantitative investment theory has emerged as a prominent and widely researched domain within the financial markets, where investors predominantly focus on discerning the intricate influences of market dynamics. In this paper, we proposed a short-term prediction-based trading strategy, which can equiponderate between return and risk, considerations while accounting for investor risk preferences. This strategy employs GM(1,1) to capture nuanced features of price dynamics in short-term intervals and update the GM(1,1) model with the latest data. Subsequently, a multi-objective planning equation is formulated to optimize asset allocations by determining the optimal holding that strikes between specific returns and risk mitigation. In the end, this work conducts a case study and sensitivity analysis using five years of gold and bitcoin price data spanning from 2016 to 2021. This empirical examination serves to affirm the efficacy and resilience of the proposed trading strategy. The case study reveals that proficient short-term price forecasting serves as a potent means to proactively mitigate risk, facilitating, judicious and objective trading practices. Moreover, it underscores the strategy’s tangible utility as a guide for real-world investment decisions.

## Introduction

Each country’s influence on investor enthusiasm has become increasingly important, as every national financial sector aims to encourage investor participation in financial activities [1–4], different strategies for different topics often lead to different results [5, 6]. In recent years, portfolio optimization and quantitative investment have emerged as crucial research fields in the financial market. With the rapid transformation of financial markets and advancements in computational technology, quantitative trading has become a widely discussed topic among investors. Quantitative trading, a strategy solely on computer algorithms, involves automated buy/sell decisions. Presently, quantitative strategies have gained broad acceptance within the investment community [7].

The core of quantitative trading decisions is the diversification of asset allocation, and the reallocation of financial resources is and fundamental principle affecting investment strategies [8]. A series of trading strategy methods originated from the Mean-Variance Model proposed by Markowitz [9], which aims to identify an optimal portfolio by considering a set of selectable asset combinations. It strives to strike a balance between returns and risks to achieve the most favorable equilibrium. In 1964, William Sharpe and John Lintner introduced the Capital Asset Pricing Model (CAPM) [10], which elucidates the relationship between risk and expected asset returns in the securities markets. The model postulates the existence of an efficient frontier, enabling investment portfolios to attain maximum returns with minimal risk. In 1976, Ross proposed the Arbitrage Pricing Theory(APT) as a model for the market’s equilibrium state [11]. Ross posited that asset returns are influenced by multiple factors, which he condensed into a comprehensive model. According to this theory, asset returns can be determined by various factors, offering investors an alternative approach to assessing the relationship between asset returns and multiple factors. The introduction of these aforementioned classic models laid the foundation for both theoretical research and practical application of quantitative trading.

Predicting stock prices has consistently remained a vital prerequisite in the realm of the stock market, as the availability of highly accurate prediction strategies can significantly enhance investor confidence. In recent years, a plethora of studies have harnessed the power of neural networks to showcase their remarkable fitting ability when employed on diverse time series data [12–14]. However, it is worth noting that neural networks typically rely on a significant number of parameters, which can result in challenges such as overfitting, slower inference speed, and potential parameter instability. These factors may limit their effectiveness in capturing the inherent characteristics of price data. In contrast, traditional machine learning algorithms often exhibit better performance in capturing the intrinsic patterns within the data itself. Grey system theory [15] is an effective approach that specifically focuses on predicting the data itself. In recent years, several studies have utilized grey system theory for predictive purposes, Faghih Mohammadi Jalali employed a first-order grey model to predict the price changes of Bitcoin, providing evidence that the grey system is capable of capturing short-term price fluctuations influenced by uncertain factors [16]. Lei introduced a novel grey forecasting model called the neural ordinary differential grey model (NODGM), specifically designed to forecast China’s crude oil consumption. This model demonstrated promising results in predicting crude oil consumption patterns [17]. Norouzi utilized the grey forecasting model to predict OPEC crude oil prices and showcased its strong applicability in this context, providing compelling evidence supporting the effectiveness and practicality of the grey forecasting model [18].

In this paper, we employ GM(1,1) as an effective forecasting model that is capable of generating efficient and reliable short-term forecasts, even when working with limited sample data and inherent uncertainty. Building upon these forecasts, we utilize a moving average strategy [19] to guide decision-making processes i.e. a buy/sell signal is issued. For trading, additionally, we employ a multi-objective planning equation to assess return and risk, thereby determining the position of each asset. To evaluate the feasibility and robustness of our proposed model, we conduct extensive simulations and perform sensitivity analysis. In conclusion, this paper conducts an example analysis focusing on the gold and Bitcoin markets from 2016 to 2021. Gold and Bitcoin are selected as representative monetary assets [20], representing robust low-risk and lot return and high-risk and high-volatility and high-return investment assets respectively [16, 21]. By examining these two assets together, we can gain a better understanding of the predictive performance and robustness of our proposed model. Furthermore, this study takes into account transaction costs during the trading process and addresses the issue of discontinuous gold trading days. By considering these real-world factors, the model aligns more closely with actual investment scenarios and provides valuable insights for guiding real investment behaviors.

The structure of the rest of this paper is as follows: In Section. Related Work, we present an overview of related works in the field. In Section. Methods, we describe the construction of the trading model. Moving on to Section. Case Study, we validate the performance of the model by applying it to datasets encompassing gold and Bitcoin. Finally, in Section. Conclusion, we summarize our findings and provide an outline for future directions within this comprehensive paper.

## Related work

### Trading strategy

Some traditional trading methods, such as [9–11] laid the basic idea of trading strategies in financial markets. Now recent researchers emphasize the use of machine learning or deep learning methods to better describe the financial market to obtain more effective market information. Kerenidis proposed a quantum algorithm for portfolio optimization, which determines the optimal risk-return and enables the extraction of the extraction optimal portfolio [22]. Ta [23] employed an LSTM (Long Short Term Memory) network to predict stock prices, complemented by various portfolio optimization techniques to enhance portfolio performance. Chunchun utilizes CNN (Convolutional neural networks) to extract more effective features from raw data for quantitative financial investment [24].

Grey system approaches have proven to be advantageous in handling sequences within markets, this is because the grey system can effectively capture the uncertainty in the financial market. Sing has introduced a novel grep prediction system for forecasting the value of various cryptocurrencies [25]. Ramezani utilized grey relational analysis to randomly select stocks from different industries listed on the Tehran Stock Exchange, this study focused on dynamic portfolio selection and its experimental implementation [26]. Trading in financial markets is similar to a game process, some trading strategies [27–30] based on deep reinforcement learning have been proposed and proven effective. In this work, we only use simple and effective MA [9] trading methods, to prove the effectiveness of our methods.

### Prediction model

Trading models rely on the accuracy of prediction models. Using models with high accuracy can greatly enhance investor confidence and help investors make decisions. Some works use neural networks for prediction. Moghar uses LSTM to build a model that can predict feature stock prices [12]. By considering the distinct characteristics exhibited by various sequences, the appropriate prediction model was meticulously constructed, encompassing the simulation of individual components. Xinrong noted that the performance ability of LSTM and CNN on stock predicting tasks, to further enhance predictive capabilities, Xinrong proposed a novel approach that combines the strengths of LSTM and CNN models [14].

The grey prediction model leverages the cumulative form of system parameters to establish rules for short-term prediction, demonstrating promising performance across diverse prediction tasks [25, 31–33]. Grey prediction models are often integrated with various other applications, Tianxiang introduced a multi-step prediction model by leveraging the combination of LSTM and GM(1,1) techniques [13]. Tang presents a novel approach to monthly electric load forecasting using a seasonal index based on grey model optimization [34]. [35] proposed a new hybrid model named FGM(1,1), which combines the grey model with fractional order accumulation, seasonal factors, Sine Cosine Algorithm (SCA) optimization, and error correction to improve prediction performance.

## Methods

### Grey theory and GM(1,1) model

Grey System Theory was proposed by Prof. Deng [15], grey system theory can mitigate the challenges posed by limited sample datasets by effectively capturing the developmental process of uncertain phenomena [36–38]. Grey predictive modeling is a prediction method based on incomplete information and a small sample dataset, which is widely used in finance, energy, materials, and other forecasting applications [25, 33, 36, 38–42]. In this paper, we use GM(1,1) as a short-term prediction model. GM(1,1) can be divided into three types: GM(1,1), new information GM(1,1), and metabolism GM(1,1).

#### GM(1,1) model

Generate an accumulation sequence from the original sequence, construct a differential equation, and derive the basic form of the GM(1,1).

$$\begin{array}{c} x^{\left(0\right)}\left(k\right)+\alpha z^{\left(1\right)}\left(k\right)=b \end{array}$$ (1)

Where −*α* is the development index, and b is the grey trigger value.

Estimate the parameters using OSL (Ordinary Least Squares) and substitute the obtained a, b into the model, resulting in the final estimate.

$$\begin{array}{c} {\hat{X}}^{\left(1\right)}\left(k+1\right)=[X^{\left(0\right)}\left(1\right)-\frac{b}{a}]e^{-\alpha k}+\frac{b}{a};k=1,2,\ldots ,n \end{array}$$ (2)

Evaluate the expression above using accumulation and reduction, and produce the grey prediction of the original sequence *x*^(0)^, as illustrated follow,

$$\begin{array}{c} {\hat{X}}^{\left(0\right)}\left(k+1\right)={\hat{X}}^{\left(1\right)}\left(k+1\right)-{\hat{X}}^{\left(1\right)}\left(k\right)=\left(1-e^{\alpha }\right)(x^{\left(0\right)}\left(1\right)-\frac{b}{a})e^{-\alpha k};k=1,2,\ldots k \end{array}$$ (3)

#### Variant of GM(1,1)

The Accuracy of GM(1,1) forecasting hinges on data distribution. Recent data is considered more influential, with new information having a significant impact. However, historical data can dampen responsiveness, causing delayed response, poor tracking, and lower accuracy. To address this problem, two GM(1,1) variants have emerged, “new information GM(1,1)” and the “metabolism GM(1,1)”. Both models attribute increased importance to recent data and perpetually update the original price sequence to incorporate incoming information. Notably, the “metabolism GM(1,1)” updates self-state, by discarding past data, thereby ensuring the model maintains a current and adaptive state, thereby enhancing its aptitude for capturing evolving trends and accommodating dynamic information dynamics.

**Fig 1:**
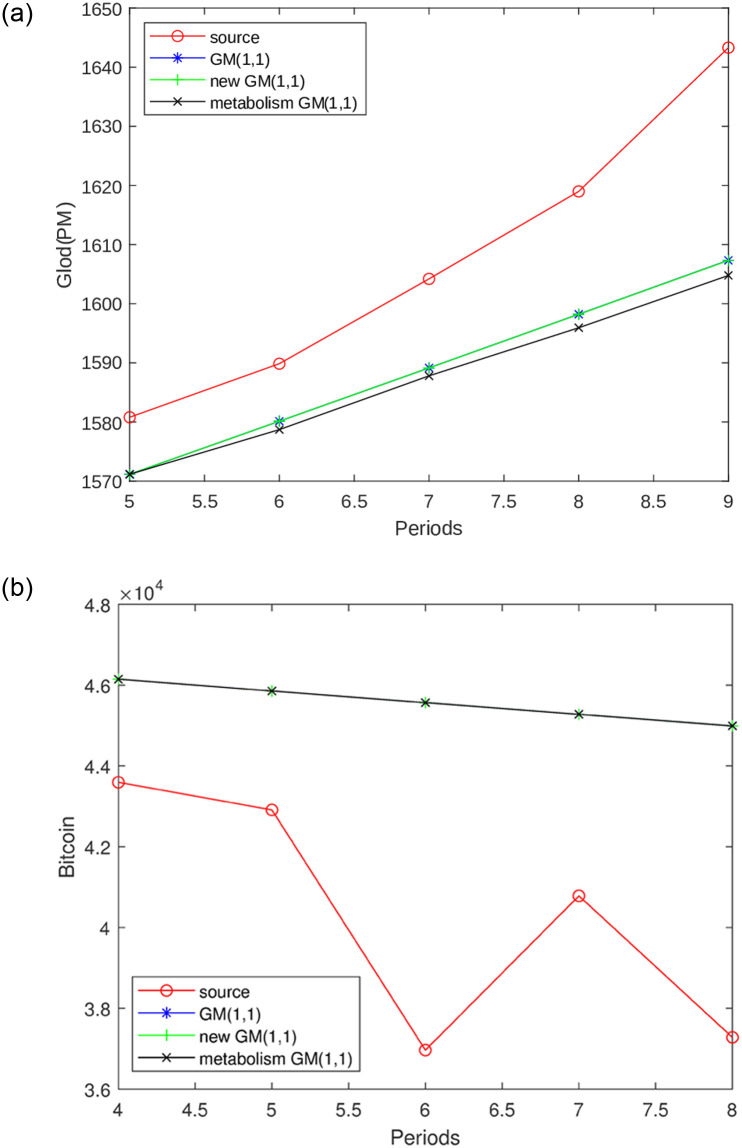
Three variants of GM(1,1) fit the original data ∘ represent original data, * denote GM(1,1), + denote new information GM(1,1), and × denotes metabolism GM(1,1). (a): Gold, (b): Bitcoin.

Three GM(1,1) models have been employed to forecast future price movements, which are subsequently assessed in comparison to the current period’s data. The model exhibiting the lowest Mean Squared Error(MSE) value is selected as the predictive model for the forthcoming period’s data. Fig 1 visually presents the outcomes of fitting these three GM(1,1) models to the original datasets.

#### GM(1,1) prediction process

**Fig 2:**
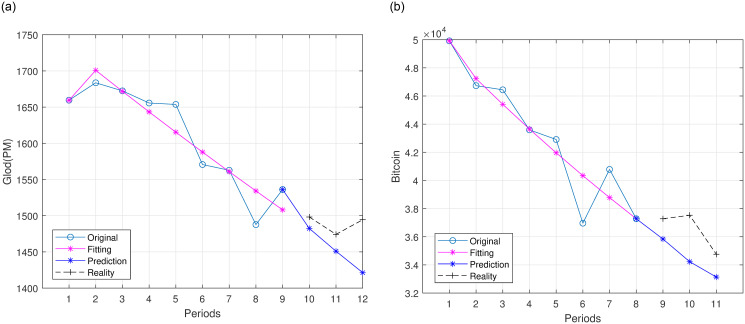
The GM(1,1) model is employed to forecast future price trends. (a): Gold, (b): Bitcoin.

Initially, we introduce the concept of a designated “windows period” for the original data. This choice is driven by the necessity within the GM(1,1) model framework to accumulate a sufficient amount of data for the attainment of efficient predictions, in this paper, the window periods are set to 4. Then this iterative process of updating the GM(1,1) model with daily refresh price data will be currently performed, facilitating the model’s continuous adapt to the dynamic market conditions. The model shall recalibrate using the updated data series, thereby yielding forthcoming near-term predictions, typically spanning three periods. A schematic illustration of this iterative procedure is shown in Fig 2.

Fig 2 offers an insightful graphical representation of the original data trend along with the predicted curve trend, with the predictive curve impeccably mirroring the trajectory of the actual data. In terms of computational, the mean relative residuals for gold and Bitcoin amounted to 1.28% and 2.81%, respectively, indicating that the predictions are highly accurate compared to the actual data.

#### Result and numerical study

The Mean Absolute Percentage Error (MAPE) stands as one of the most widely employed metrics for assessing forecast accuracy. Given its unitless nature, MAPE can provide valuable insights into the magnitude of forecasting errors, making it a versatile tool for error evaluation [16, 43, 44]. Its calculation is as follows,

$$\begin{array}{c} \text{MAPE}=\frac{1}{N}\sum_{i=1}^{N}\frac{|A_{t}-F_{t}|}{A_{t}} \end{array}$$ (4)

Where *N* is number of data, *A*_*t*_ denotes *t*^*th*^ data, *F*_*t*_ denotes *t*^*th*^ prediction data.

The GM(1,1) models achieved a commendable level of accuracy for pre, in numerical terms, the MAPE for gold is 0.48%, and for Bitcoin stands at 2.14% when considering all the examined periods. underscoring their robust performance in forecasting price movements for these assets. Table 1 displays the predicted and actual values for both gold and Bitcoin during the partial periods.

**Table 1:** Partial prediction result for both gold and bitcoin.

Current	Gold(PM)		Bitcoin($)	
Periods	Prediction	Actually	Prediction	Actually
1	1659.600	1659.600	49922.520	49922.520
2	1700.907	1683.650	47239.734	46736.580
3	1671.922	1672.500	45412.005	46441.640
4	1643.430	1655.700	43654.992	43596.240
5	1615.424	1653.750	41965.959	42912.190
6	1587.895	1570.700	40342.275	36964.270
7	1560.835	1562.800	38781.412	40784.320
8	1534.236	1487.700	37280.940	37280.350
9	1508.091	1536.200	-	-
Prediction	Gold(PM)		Bitcoin($)	
1	1482.391	1498.200	35838.522	37528.300
2	1450.933	1474.250	34230.464	34754.540
3	1421.313	1494.400	33137.130	38728.590

### Trading strategy based on price dynamics

#### Trading strategy

This paper employs a classical trading strategy. Known as Moving Average Theory (abbreviated as MA). The Moving Average Theory can be traced back to the work of Shiller in 1980, who observed that asset prices tend to revert to their mean values over extended time intervals [19]. Currently, the Moving Average Theory has evolved into a well-established framework and is utilized to represent the average stock price average, calculation see Eq 5.

$$\begin{array}{c} \text{MA}=\frac{c_{1}+c_{2}+\ldots +c_{N}}{N} \end{array}$$ (5)

Where MA denotes the current price’s average line, *N* represents the number of calculation periods, and *C*_*N*_ denotes the price observed on the *N*^*th*^ day.

The MA strategy under consideration comprises both short-term and long-term moving averages (MA). The short-term MA is used to capture the immediate price behavior, typically reflecting market fluctuations over a brief temporal interval. Conversely, the long-term MA encapsulates the more protracted price trends, offering insights into the average transaction costs sustained over a more extended timeframe. Historically, prior research has underscored the notion that once a market trend takes shape, it tends to persist over a defined period. Specifically, when the short-term MA intersects with the long-term MA, it signifies that the recent uptrend in prices surpasses the long-term average trend. This occurrence is interpreted as a buy signal, suggesting a prospective price increase in the feature. Conversely, when the short-term MA crosses below the long-term MA, it indicates an impending price decrease, indicating a sell signal.

#### Multi-objective planning equation

In real-world problem-solving scenarios, the need arises to contemplate not solely the optimal resolution for a single objective but also the situations where multiple objective values necessitate simultaneous optimization [8, 45]. Particularly in the realm of practical investments, investors face the imperative of striking a delicate equilibrium between maximizing returns and mitigating risks. Consequently, this paper endeavors to address the intricate interplay between these dual objective investment portfolio models. This model seeks to attain a balance of limited profit and minimizing risk, while also accommodating the investor’s risk preference level.

*Objective function*. Within the realm of financial markets, the fundamental aim of investors resides in the pursuit of asset appreciation while simultaneously endeavoring to mitigate trading risks. In light of this point, this paper endeavors to formulate a multi-objective optimization model that harmonizes the dual imperatives of maximizing returns. Consequently, a positive objective is to aptly steer the model towards the attainment of enhanced returns. The formulation is as follows.

$$\begin{array}{c} \text{max}\;w=h_{0}+\sum_{j=1}^{2}p_{t}h_{j} \end{array}$$ (6)

Where *h*_0_ denotes the amount of cash held, *p*_*t*_ denotes the *t*^*th*^ dally price, and *h*_*j*_ denotes the holdings of gold and Bitcoin, respectively (*j* = 1, 2).

In the trading process, investors are prudent to seek the minimization of risk to the fullest extent. However, if investors opt to maintain their holdings solely in cash within the market, the risk is decreased to its nadir, while this strategy will result in an absence or returns on their investments. Such an outcome, conspicuously incongruous with the fundamental tenet of profitability, fundamentally contravenes the investors’ original intent. Generally, investors consistently aspire to strike a balance between returns and risk. Consequently, an additional optimization objective is introduced, focusing on the management of the number of holdings for each asset to effectively mitigate risk. The objective function at risk minimization is as follows,

$$\begin{array}{c} \text{min}\;r=\sum_{j=1}^{2}\rho_{j}p_{t}h_{j} \end{array}$$ (7)

Where *h*_*j*_ represents the holdings of gold and Bitcoin, respectively. *p*_*t*_ denotes current day price, signifies risk factor, which measures the risk level of gold and Bitcoin over a window period. In this paper, the variance is used to calculate this value, for example, if the price of Bitcoin is more volatile over a period of time, then the risk factor weights are larger. This parameter is automatically calculated by the model in programming.

For the multi-objective planning equation, in the research, there are two common treatments for the objective function, which are weighted optimization and staged optimization [46]. In this paper our focus lies on the utilization of weighted optimization, we use weighted optimization, which is uniformly converted to a max problem, and after weighted treatment, we get the final objective function as shown in Eq 8

$$\begin{array}{c} \text{max}\;w=[\mu (h_{0}+\sum_{j=1}^{2}p_{t}h_{j})+(1-\mu )(-\sum_{j=1}^{2}\rho_{j}P_{t}h_{j})] \end{array}$$ (8)

In this paper, it is assumed that the variable *μ* represents the weighting coefficient, which quantifies the trade-off between gains and losses. The value of *μ* falls within the interval [0, 1]. Moreover, this parameter serves as an indicator of investors’ risk preferences. Specifically, when *μ* assumes smaller values (like 0.1), it signifies a diminishing emphasis on return targets and greater consideration of risk reduction, characterizing a more conservative investor profile. In this paper, we assume that investors exhibit a balanced profile thus *μ* is set as 0.5.

*Constraint condition*. In this paper, we control transaction volume through the application of a planning equation. This equation plays a pivotal role in ascertaining the profitability of each transaction, considering both the elements of risk and return, while also factoring in the transaction commission i.e., transaction costs. Transactions are only executed when anticipated benefits from the current trade exceed the transaction costs. In essence, trades are conducted only when the model perceives the current time interval as being profitable. Furthermore, the model prohibits short selling, meaning it is not possible to acquire assets beyond the current total asset value. And the holding of the three asset types must remain non-negative, in accordance with real-world constraints. To summarize, the constraint conditions for the multi-objective planning model are as follows:

$$\begin{array}{c} \text{st}.\;\{\begin{array}{cc} & h_{0}+\sum_{j=1}^{2}p_{t}h_{j}-\sum_{j=1}^{2}x_{j}\alpha_{j}\geq x_{j}\times p_{t}\\ & \sum_{j=1}^{2}x_{j}\times (|p_{t}-{\hat{p}}_{t+1}|)>\sum_{j=1}^{2}\alpha_{j}p_{t}x_{j}\\ & h_{i}\geq 0 \end{array} \end{array}$$ (9)

where, *x*_*i*_ represents transaction volume for gold and Bitcoin, respectively. $\hat{p_{t+1}}$ denotes average value from GM(1,1) prediction. *α*_*j*_ represents the transaction commission ratio for gold and Bitcoin, respectively, representing the transaction costs.

Notably, we mentioned that the trading day is not consistent in the gold market, the price of gold on a non-trading day remains static, and due to the existence of transaction costs, the model does not perceive such situations as profitable and would not transaction in the gold market, which solves the problem of inconsistent trading days.

## Case study

### Why choice gold and bitcoin

Decision-making constitutes a primary concern for both leaders and investors [3, 47]. Investors exhibit a dedicated commitment to the meticulous examination of the intricate interplay between conventional and emerging digital assets [48]. Investors, in pursuit of rational asset allocation, endeavor to optimize their portfolios, and they achieve this by employing hedging instruments that provide a relatively robust foundation [49]. Some empirical investigations [49, 50] have found that gold and Bitcoin exhibit a non-linear link with low correlation, making gold and Bitcoin a diversified pair of hedging assets in a portfolio. In the case study, we use gold and Bitcoin as the research objects.

Within the realm of precious metals, gold stands as reverence and prestige. The gold market is subject to the dynamics of speculation and volatility due to its growing storage value and fields of use [42, 51]. Cryptocurrency is a decentralized digital currency [52], that relies on a decentralized blockchain-based transaction system [50, 53]. It empowers users to securely and anonymously transfer and store value within the network. Presently, Bitcoin stands as a global frontrunner among cryptocurrencies.

In the section on empirical analysis, this paper defines three different asset types: Cash, Gold, and Bitcoin. Assuming the initial capital is $1000, the initial state is set as [1000, 0, 0], which is the starting state of the program simulation. In addition, the model also considers fees in transactions. The transaction fee for Gold is assumed to be 1% of the transaction amount, and for Bitcoin, it is 2%. Each transaction should deduct the cost at the time of the transaction, and only the profit obtained should be considered. If there is no transaction fee, the model will continue to conduct transactions, which is not realistic. At the same time, this paper also discusses the trading days of Gold in the multi-objective optimization model, which makes the trading model more realistic.

### Data description

In this work, the research foundation is established upon the daily trading prices of both gold and Bitcoin markets during the periods spanning from 2016 to 2021. The gold data originates from the London Bullion Market Association (LBMA), a globally recognized and authoritative entity within the sphere of precious metal valuation agencies. Specifically, we procure the daily closing price of one ounce of gold denominated in USD from the official LBMA website. While the Bitcoin price data is sourced from the website https://data.nasdaq.com/data/BCHAIN/MKPRU-bitcoin-market-price-usd. This website offers a user-friendly interface for an intuitive display of daily Bitcoin prices and provides the capability to download entire datasets for comprehensive analysis.

It should be noted that gold trades on specific trading days, while Bitcoin can be traded almost continuously.

### The quasi-exponential testing of data

From the original data series, the application of GM(1,1) is limited to data series adhering to the quasi-exponential law. The smoothness ratio of the initial sequence *x*^(0)^ is defined as Eq 10

$$\begin{array}{c} \rho \left(k\right)=\frac{x^{\left(0\right)}\left(k\right)}{x^{\left(1\right)}\left(k-1\right)} \end{array}$$ (10)

Where *k* represents the *k*^*th*^ period data, for ∀*k*, *ρ*(*k*) ∈ [*a*, *b*] and interval length *δ* = *b* − *a* < 0.5, then the cumulated data exhibits characteristics indicative of a quasi-exponential law.

In practical computations, it suffices to ascertain that over 80% of the data points possess a smoothing ratio of less than 0.5 to conclude that the cumulative data adheres to the quasi-exponential law and can be used with the GM(1,1) model.

**Fig 3:**
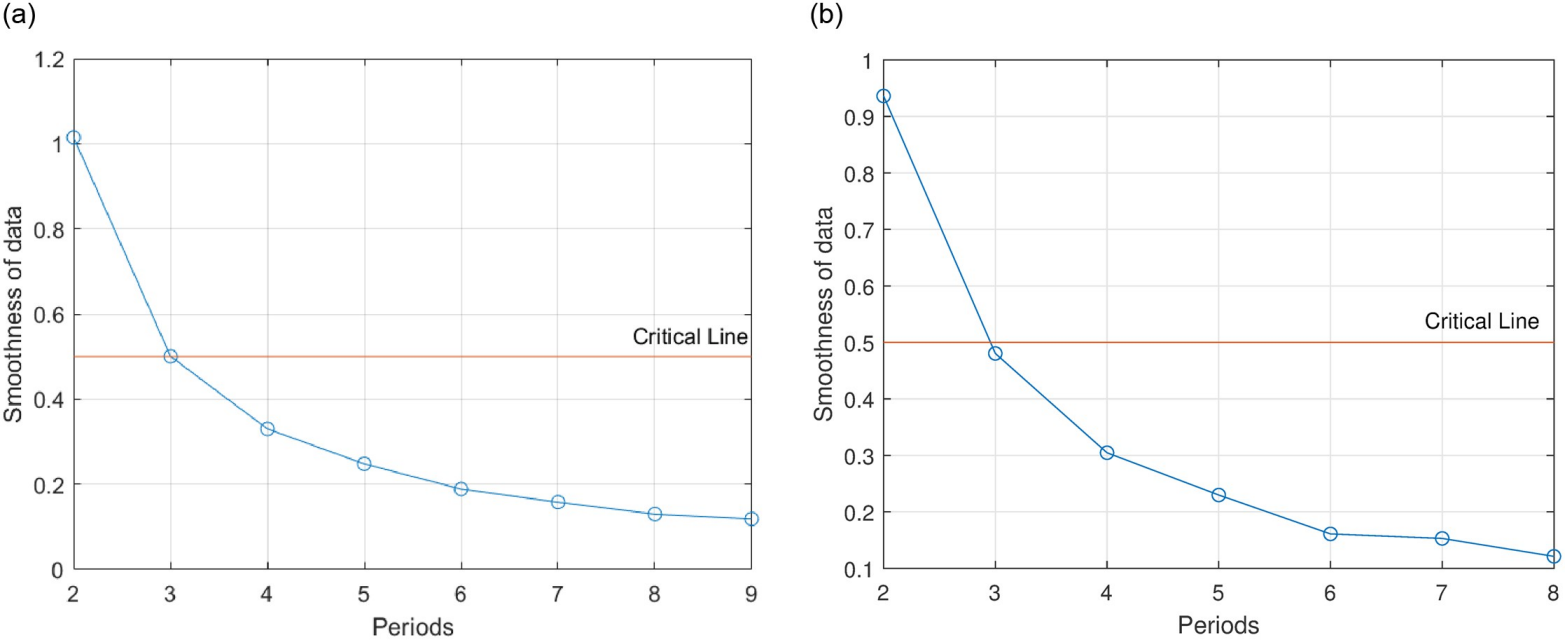
Quasi-exponential testing of data series. (a): Gold, (b): Bitcoin.

Fig 3 demonstrates that starting from the third period of data, both the gold and Bitcoin data sequences exhibit a smoothing ratio consistently below 0.5. moreover, the proportion of periods with a smoothing ratio below 0.5 exceeds 80%. Consequently, the data sequence has successfully passed the quasi-exponential test, thereby affirming their suitability for modeling using the grey prediction model.

### Model simulation

**Fig 4:**
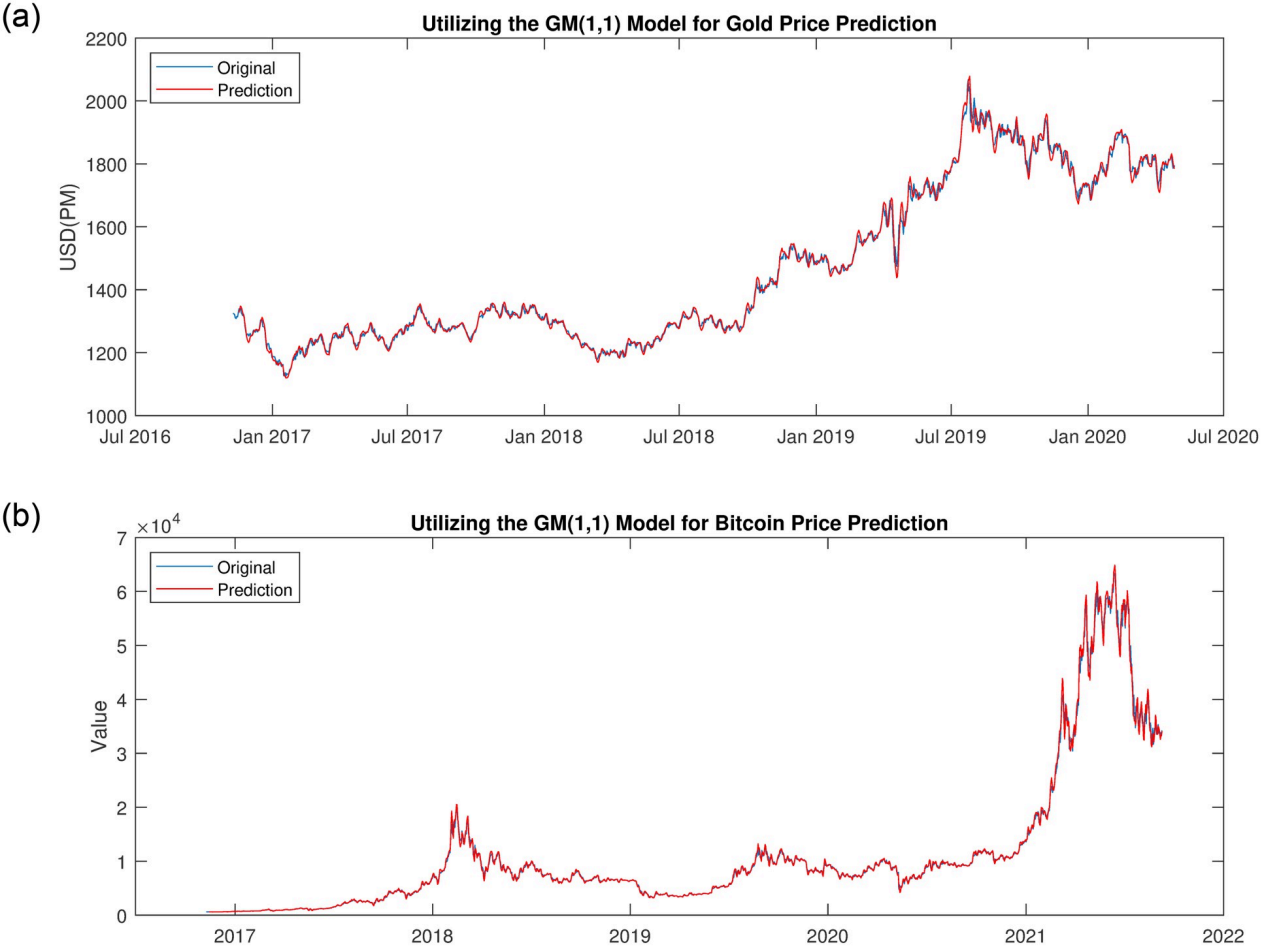
Efforts in fitting gold and bitcoin price data. (a): Gold, (b): Bitcoin.

Utilizing the GM(1,1) model, the price sequence data is cumulated, yielding a first-order accumulated sequence. Parameters are estimated based on Eq 1, and these estimations are subsequently substituted into Eq 2 to derive the accumulated data for forthcoming periods. Subsequently, the data is de-cumulated employing Eq 3 to derive predicted data for feature periods. This process employs three distinct GM(1,1) models in Section. Variant of GM(1,1), and the model with the lowest MSE is chosen for forecasting future data points. Upon the arrival of re-fresh price data, the model is updated to adapt to this fresh information. This iterative approach leads to the fitted data displayed in Fig 4.

As depicted in Fig 4 both gold and Bitcoin exhibit a parallel trend in line with actual data fluctuations. However, the models’ performance in capturing intricacies in Bitcoin’s behavior during periods of pronounced volatility falls short. To mitigate the risk of overfitting and curb excessive trading, the model intentionally incorporates incomplete fitting strategies in regions characterized by significant volatility.

The process commences with the derivation of price fitting data through the GM(1,1) model in Section. Grey Theory and GM(1,1) Model. Subsequently, trading determinations are executed by employing the moving average strategy outlined in Section. Trading Strategy. Following this, a planning equation that effectively balances return and risk with a dual-factor consideration is employed to gauge risk and return levels. Ultimately, the entire trading procedure is visualized using Python.

**Fig 5:**
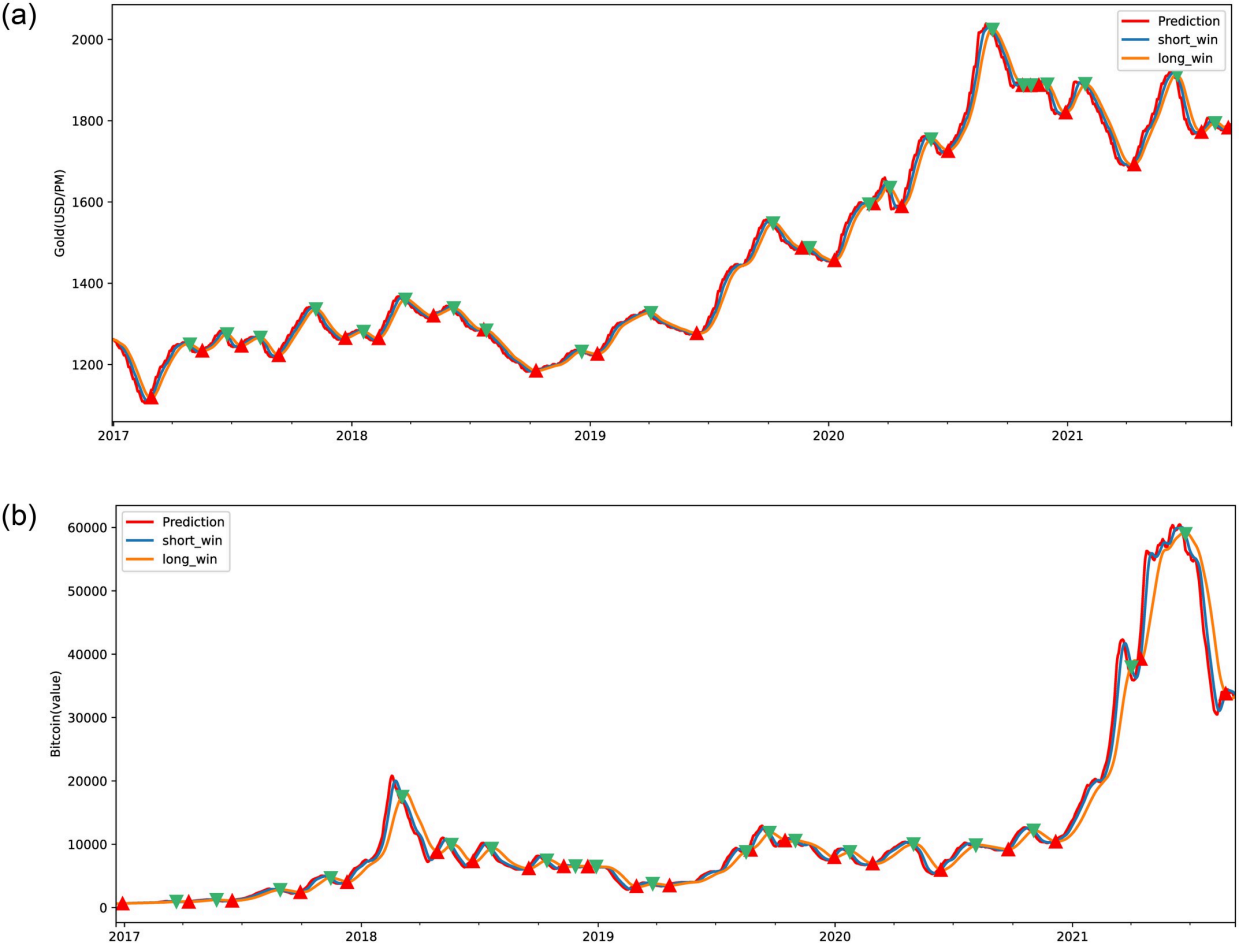
The trading process for both gold and bitcoin. Where ▴ represents a buy signal, ▾ denotes a sell signal. (a): Gold, (b): Bitcoin.

The full trading process for both gold and Bitcoin is shown in Fig 5. Gold displays a generally upward trajectory, particularly during the period from 2018 to 2020, as several countries began to view gold as a strategic reserve, leading to increased demand and subsequent price rises. In contrast, Bitcoin exhibits a gradual uptrend, albeit with periods of heightened volatility. Notably, around May 2021, Bitcoin achieved a peak price of $64,544.44 before experiencing a decline. Remarkably, the model not trading on Bitcoin even after identifying its downtrend, opting instead to continue exploring opportunities in the gold market.

**Fig 6:**
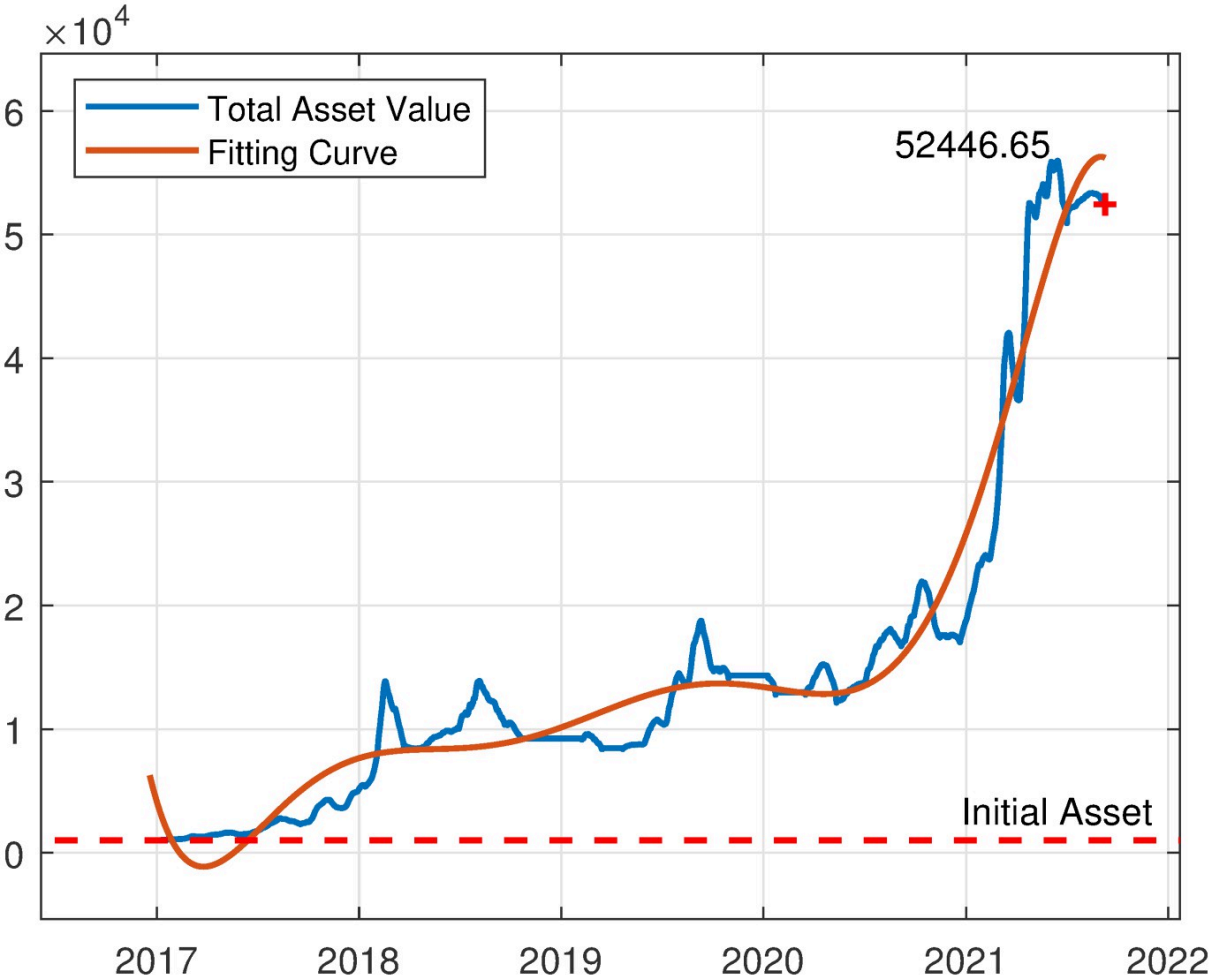
Total asset value dynamics with an initial cash reserve of $1000.

As shown in Fig 6. After the completion of the model simulation, the total value of held currency assets is $52,466.65. Starting with an initial capital of only $1000, these assets have grown to approximately 5.2 times the initial investment over approximately five years, with an annualized return rate of 11.10%. This demonstrates the feasibility of the short-term price prediction model developed in this study. If traders were to invest more initial capital and employ more detailed trading strategies, even more remarkable returns could be achieved.

### Sensitivity analysis

The aforementioned paragraph underscores the incorporation of practical trading considerations into this case study. Specifically, transaction costs accompany every transaction and a variant of assets with different costs. These varying transaction costs may exert distinct influences on the model’s results, to demonstrate the robustness of the model, this work embarks on a sensitivity analysis of transaction costs.

$$\begin{array}{c} \alpha_{i}=\alpha_{\text{i}}-0.005,\;\alpha_{i}=\alpha_{i}+0.01\;\left(i=1,2\right) \end{array}$$ (11)

**Fig 7:**
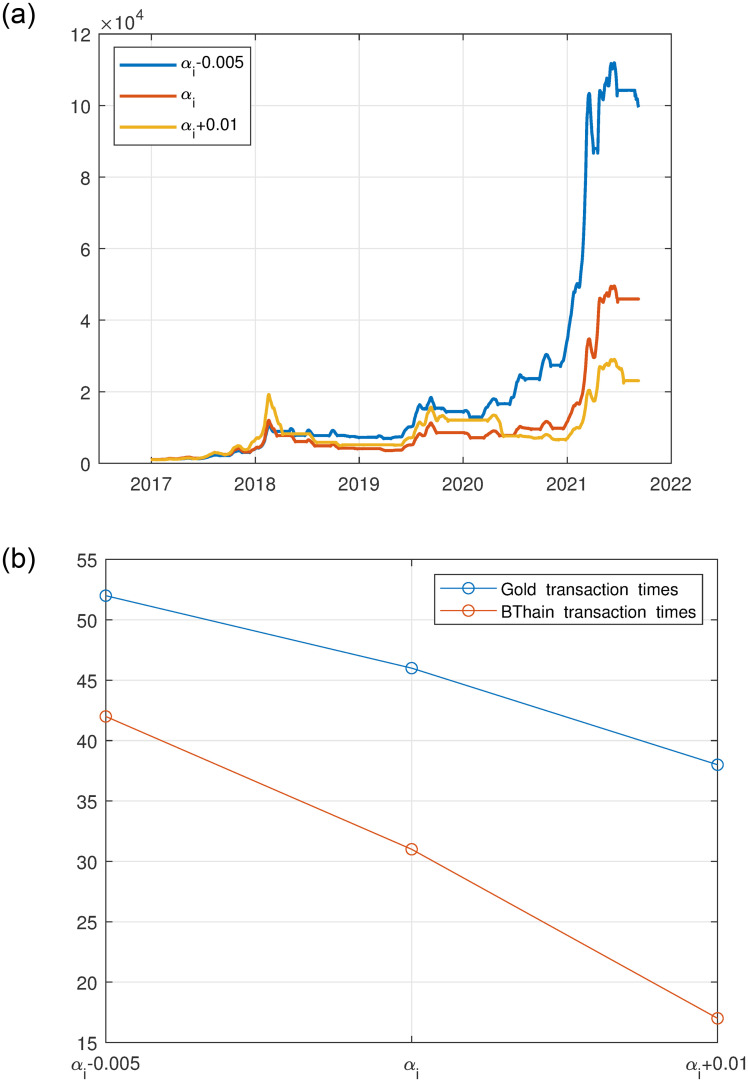
The impact of fees on total asset value, and transaction times. (a): impact of different fees on total asset value, (b): the impact of different fees on the transaction times.

Explore the impacts of different fee rates on the total asset value and the frequency of transactions for both gold and Bitcoin, the result is shown in Fig 7.

Fig 7 illustrates the effect of varying transaction fees on the total asset value and transaction times. It can be soon that when the fee rate increases by 0.01, the model’s final asset worth is roughly $22,000, accompanied by transaction times drop, from 46 to 38 times for gold and from 31 to 17 times for Bitcoin. On the other hand, when the fee rate decreases by 0.05, the profitability of the model increases significantly. The total asset worth obtained from the simulation is approximately $110,000 which is a 90.7% increase in profitability compared to the original total value and is accompanied by a transaction times incrassation, from 46 to 52 times for gold and from 31 to 42 times for Bitcoin.

The whole process can be depicted as follows. The increase in the fee rate per trade rises, resulting in fewer profit opportunities, and the model becomes cautious and therefore reduces the transaction times. However, despite the increase in transaction costs, the trading strategies are still able to identify profit opportunities to increase the total asset worth. Meanwhile, Fig 7 shows that as fee rates ascend, there is a concomitant decline in the number of trades executed for both gold and bitcoin, with bitcoin exhibiting a more pronounced reduction. This observation can be attributed to Bitcoin’s heightened volatility and overall risk profile, prompting the model to reallocate investments toward a more stable asset, such as gold. Conversely, in scenarios marked by descending fee rates, transaction costs diminish, profit opportunities expand, and the model can effectuate a more diversified asset portfolio with increased adjustments, culminating in an augmented volume of trades. Ultimately, this leads to investors realizing nearly twice the returns in environments characterized by lower transaction costs.

## Conclusion

Interest inefficient investment techniques have been fast expanding among investors in financial markets, both individual and corporate stakeholders. Investors seek optimal trading strategies that can enhance the efficiency and dependability of their trade executions. Previous research has concentrated on complicated stock indicators and constructed an intricate model, which exhibits reduced sensitivity to short-term price volatility and often exhibits limitations in terms of capital adjustment flexibility and the ability to facilitate swift decision-making. Therefore, this work proposes a trading strategy based on short-term price dynamics, which employed GM(1,1) to capture price dynamics in short-term intervals, and a multi-objective planning equation is formulated to optimize asset allocations, taking into account both return and risk factors. In the case study, we conducted a comprehensive evaluation of the model’s efficacy within the gold and Bitcoin markets, spanning from 2016 to 2021. The results revealed that the GM(1,1) model consistently demonstrated a high level of forecasting performance in financial markets, with MAPE of 0.48% for gold and 2.14% for Bitcoin our trading strategies effectively identified anticipated returns and associated risks while successfully capitalizing on profitable opportunities to optimize the allocation of diverse assets, culminating in a total asset valuation of $52,466.65. Furthermore, we incorporated real-world transaction costs into our analysis, meticulously examining the influence of commission rates on the total asset value and the transaction times. This meticulous consideration underscores the robustness of our model, rendering it not only empirically robust but also highly instructive for practical applications in real-world financial contexts.

Feature work will focus on improving the design of trading strategies and exploring more efficient trading methods, and through these efforts, we aim to further enhance the performance and reliability of the model.
